# Adverse device reaction: Chin decubitus

**DOI:** 10.1111/ggi.14831

**Published:** 2024-02-13

**Authors:** Ami Schattner, Ina Dubin

**Affiliations:** ^1^ Department of Medicine Laniado University Hospital, Sanz Medical Center Netanya Italy; ^2^ Adelson School of Medicine Ariel University Ariel Israel; ^3^ Faculty of Medicine Hebrew University Hadassah Medical School Jerusalem Israel

Dear Editor,

A 95‐year‐old community‐dwelling woman became poorly responsive with refusal to feed over 2 days and was referred to the hospital on the initiative of her caretaker. She was a frail woman with a history of multimorbidity, including severe Alzheimer's dementia and recurrent falls. Two weeks before she fell, she sustained a C1 vertebral fracture and was discharged home with a neck support collar, remaining bedridden since. Before admission, she gradually deteriorated without fever or complaints of pain, until she became stuporous. On examination, the collar was removed, revealing a 3‐cm round stage 2 chin pressure ulcer with purulent discharge and surrounding skin–soft tissue infection (Fig. 1). She was afebrile and stable with otherwise unremarkable examination. Chest X‐ray and basic tests were normal, other than increased blood urea nitrogen (24.4 mg/dL, N 8–23) and C‐reactive protein (6.9 mg/L, N 0–5). She improved with intravenous fluids and topical wound care, and was discharged back home with an Aspen cervical collar, restricting motion while providing broad contact below her chin ulcer.

**Figure 1:**
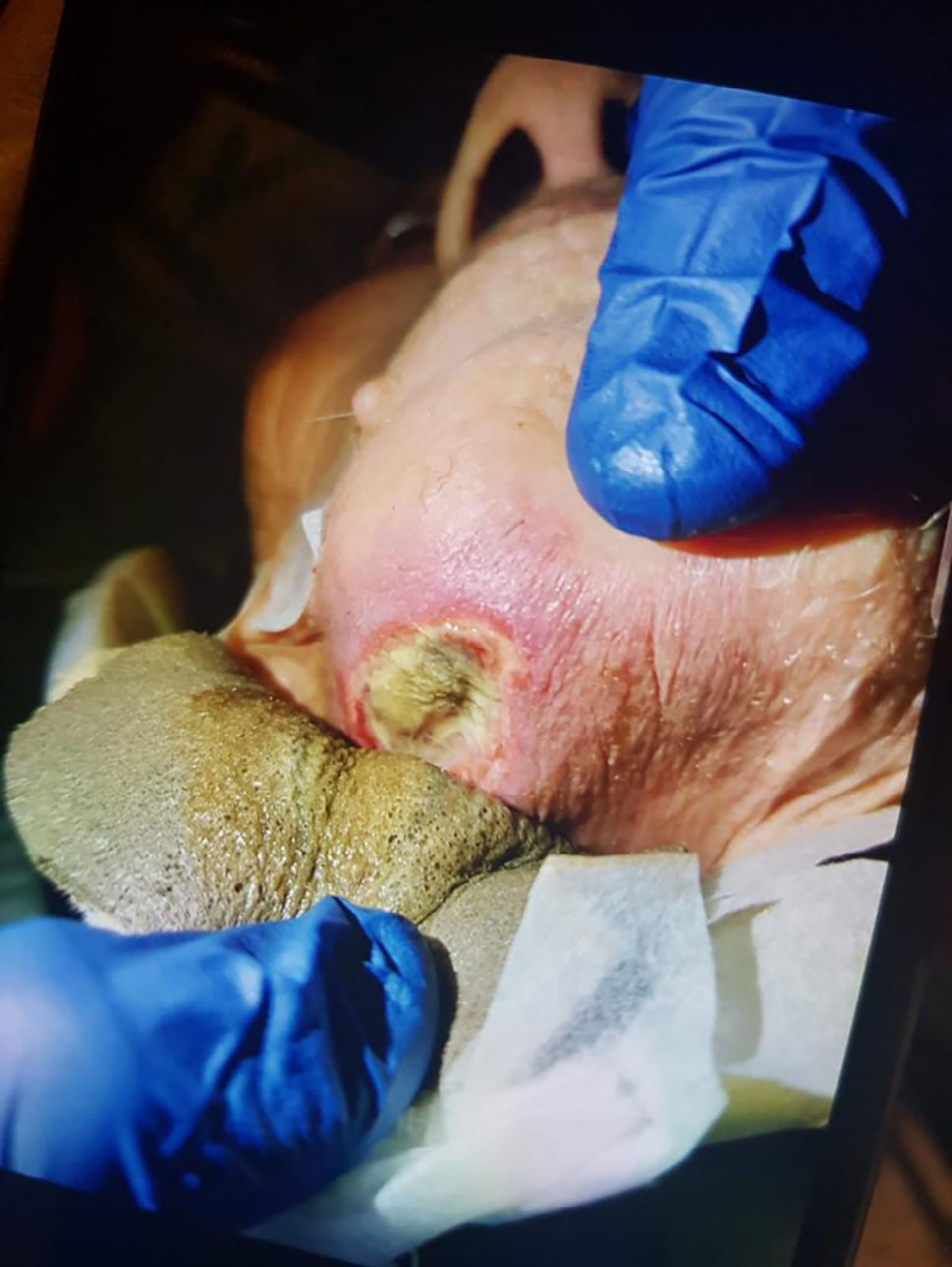
A substantial decubitus ulcer was discovered under this older adult patient's cervical collar, 2 weeks after placement – a not widely known iatrogenic complication in older ambulatory patients.

Pressure ulcers often develop in bedridden older adults, mostly over the sacrum/coccyx, heels and hips, strongly associated with immobility, malnutrition and incontinence. They have been linked with a twofold increase in length of hospital stay, fourfold increase in costs and a fivefold increase in mortality.^1^

Medical device‐related pressure injuries (MDRPI) are less well known, but have been reported with respiratory devices (most common: endotracheal tubes or noninvasive ventilation masks); urinary catheters; nasogastric tubes; and underneath straps, cervical collars and splints. They are now acknowledged as important patient safety events and nursing‐quality indicators,^2^ although this mainly applies to intensive care unit (ICU) patients where the reported prevalence of MDRPI (skin or mucous membranes) can reach 27.9–34%.^3^ Thus, most studies identified in a recent meta‐analysis involved younger trauma patients,^2^ and pressure ulcers under cervical collars used long term for cervical spine immobilization were usually limited to younger ICU trauma patients.^2^, ^4^

However, iatrogenic pressure ulcers in atypical locations have also been reported in older adults, and one prospective study noted an incidence of 5.7% among chronic geriatric inpatients.^5^ Iatrogenic pressure ulcer on the chin is unusual in older adult patients, especially when not associated with ICU admission. The increasing use of the prone position with acute respiratory distress syndrome and COVID‐19 might increase the risk of pressure injury on the chin, independent of the use of devices.

Risk factors for MDRPI include patient‐related factors (e.g. older age, diabetes, edema), treatment‐related factors (e.g. use of vasoconstrictors, prone position) and device‐related factors (e.g. longer duration of use, number of devices). Typically, a patient has several MDRPI risk factors (e.g. 2.8 ± 1.5 per patient in one study). Risk factors in older patients are similar, but aging changes in the skin, and frequent immobility, malnutrition, compromised circulation and clouded sensorium make them significantly more vulnerable.

Our report is a reminder that, albeit unusual, pressure ulcers can develop in atypical locations in older adults, with or without ICU admission, adding to the wide spectrum of healthcare‐associated harm,^6^ and highlighting the need to always remove and examine under every medical device of older patients seen in the emergency department.

## Data Availability

Data sharing is not applicable to this article as no new data were created or analyzed in this study.
